# Nonlinear Relationships of Fibrin Network Structure as a Function of Fibrinogen and Thrombin Concentrations for Purified Fibrinogen and Plasma Clots

**DOI:** 10.64898/2026.01.22.700990

**Published:** 2026-07-29

**Authors:** Can Cai, Zezhong Zhang, Arezoo Nameny, Daniel Nelli, Keith Bonin, Brittany E. Bannish, Nathan E. Hudson, Glen Marrs, Stephen R. Baker, Martin Guthold

## Abstract

**Background:**

Fibrinogen levels are associated with bleeding disorders and thrombotic disease. Thrombin converts fibrinogen to fibrin, producing the load-bearing fibrin scaffold that governs clot mechanics and transport.

**Objective:**

Quantitatively map how initial fibrinogen and thrombin concentrations, [ *Fgn* ] _0_ and [ *T* ℎ *r* ] _0_ , determine fibrin architecture in human plasma and a purified fibrinogen system.

**Methods:**

Scanning electron microscopy was used to quantify single-fiber morphology—fiber diameter and branch-to-branch segment length from a standardized sample-preparation protocol. Confocal microscopy was used to quantify network architecture—projected fiber density and pore/bubble size.

**Results and Conclusions:**

Across plasma and purified systems, fibrin structural parameters were quantitatively captured by compact multiplicative scaling laws of the form *Y* = *k* [ *Fgn* ] _0_ *^α^* [ *T* ℎ *r* ] _0_ *^β^* . Unlike prior studies, which examined narrower condition ranges without establishing predictive equations across a systematic fibrinogen–thrombin concentration matrix, this framework defines distinct, quantitative roles for fibrinogen and thrombin in fibrin assembly. The magnitudes and signs of the exponents α and β indicate that thrombin primarily controls individual fiber growth kinetics, strongly shortening branch-to-branch segment length and modestly thinning fibers, whereas fibrinogen primarily controls space filling, strongly increasing fiber density, reducing pore/bubble size, and thickening fibers. For matched [ *Fgn* ] _0_ and [ *T* ℎ *r* ] _0_ , compared to plasma clots, purified fibrinogen formed denser networks with thinner and shorter fibers, suggesting that the plasma biochemical environment partially inhibits thrombin activity. Fiber length analysis further suggests that each thrombin molecule nucleates one fiber segment. Together, these parameterized scaling relations provide a predictive quantitative framework linking clot composition to fibrin microstructure in plasma and purified fibrinogen clots.

## Full Text Availability

The license terms selected by the author(s) for this preprint version do not permit archiving in PMC. The full text is available from the preprint server.

